# Long-term headaches in children with idiopathic intracranial hypertension—a 10 years follow-up

**DOI:** 10.3389/fped.2026.1726347

**Published:** 2026-05-14

**Authors:** Jacob Genizi, Moran Cymbrowicz, Lilach Shemer-Meiri, Noam Ganz, Ayellet Sadeh, Rony Cohen, Rajech Sharkia, Abdelnaser Zalan, Salam Abd El Karim Murad, Esther Ganelin-Cohen, Muhammad Mahajnah

**Affiliations:** 1 The Ruth and Bruce Rappaport Faculty of Medicine, Technion—Israel Institute of Technology, Haifa, Israel; 2 Pediatric Department, Bnai Zion Medical Center, Haifa, Israel; 3 Pediatric Department, Carmel Medical Center, Haifa, Israel; 4 Pediatric Neurology Unit, Carmel Medical Center, Haifa, Israel; 5 Department of Pediatric Neurology, Schneider Children’s Medical Center of Israel, Petah Tikva, Israel; 6 Gray Faculty of Medicine and Health Sciences, Tel Aviv University, Tel Aviv, Israel; 7 Unit of Human Biology and Genetics, The Triangle Regional Research and Development Center, Kafr Qara, Israel; 8 Unit of Natural Sciences, Beit Berl Academic College, Beit Berl, Israel; 9 Baqa College, Baqa Al-Gharbia, Israel; 10 Child Neurology and Development Center, Hillel Yaffe Medical Center, Hadera, Israel

**Keywords:** headache outcome, idiopathic intracranial hypertension, IIH, pseudotumor cerebri, PTC

## Abstract

**Introduction:**

Idiopathic Intracranial Hypertension (IIH) is characterized by increased intracranial pressure, often accompanied by headaches, which were traditionally expected to resolve with pressure reduction. The aim of present study was to assess the prevalence of persistent headache and quality of life 10 years after the initial diagnosis of IIH and to determine the factors, at the time of the diagnosis, that predict associated with these ongoing symptoms.

**Materials and methods:**

In a cross-sectional multicenter cohort study, patients diagnosed with IIH (all included patients met the Friedman criteria) in 2007–2012 were contacted by telephone at least 10 years after initial diagnosis, in 10.24–12.24. Participants responded to two questionnaires: the HIT-6, to evaluate headache burden, and the WHOQOL-BREF quality-of-life questionnaire.

**Results:**

Of the 98 patients, 81 were recruited for the study. Mean age at IIH diagnosis was 13 (±2) years and mean age at follow-up was 24 (±5) years. Sixty-six percent (54/81) were females. Of these, 47 patients (58%) were experiencing headaches at the time of follow-up, with no significant difference between the sex. Relative to the no-headaches group, those with headaches at follow-up had higher BMI (32 vs. 25, *p* = 0.041) and lower quality of life (QoL). Within the headaches at follow-up group, those with more significant headache burdens (HIT-6 levels 3–4) were older, had higher BMI, and had lower QoL relative to those with lower headache burdens (*p* = 0.020, *p* = 0.006, *p* = 0.010 respectively).

**Conclusions:**

A large proportion of patients with IIH continue to suffer from headaches many years after initial diagnosis, with negative effects on their quality of life.

**Clinical Trial Registration:** ClinicalTrials.gov, Identifier NCT06581185.

## Introduction

Idiopathic Intracranial Hypertension (IIH) is an uncommon syndrome characterized by increased intracranial pressure with no clear cause, leading to pressure on the optic nerve and headaches (1). Recent advances have refined understanding of IIH pathophysiology, with evidence pointing toward a multifactorial process involving impaired Cerebrospinal Fluid (CSF) absorption, altered cerebral venous outflow, and metabolic–hormonal influences (2, 3).] In children the estimated annual incidence is 0.6–0.9 per 100,000, with epidemiological features differing from adults. Prepubertal patients show a lower prevalence of obesity and weaker female predominance, whereas post-pubertal adolescents resemble adults in risk profile.

The most widely accepted diagnostic criteria are those proposed by Friedman el al. (4), requiring symptoms IIH such as headaches or vomiting, papilledema along with normal brain imaging, except for features suggesting of IIH. Patients should have a normal neurology examination, except 6th nerve palsy, and elevated lumbar puncture opening pressure with normal cerebrospinal fluid (CSF) composition (4). For pediatric patients, updated consensus recommendations (5) emphasize a slightly higher normal CSF opening pressure threshold (≥280 mm H_2_O in children and 250 mm H_2_O if the child is not sedated and not obese) and the importance of urgent ophthalmological evaluation. Most children with IIH experience only mild visual acuity loss and visual field defects. Quality of life was reported in 1 study among children with IIH. It was lower than the general population but similar to children with headaches (6). The condition is usually self-limited disease, with acetazolamide used as first-line therapy to lower ICP by reducing CSF production (7). Severe or refractory cases may require CSF shunting. IIH recurrence occurs in approximately one-third of pediatric cases, typically within 1 year after treatment cessation in prepubertal children, and within 3 years in older patients (8).

While short term improvement is common as Yamamoto et al. (9) reported IIH resolution in 50% of cases within 1 week, 57% at 1 month, and 69% at three months following initial treatment. The long-term course of headache in children with IIH remains poorly understood. The present study aims to assess the long-term outcome regarding headaches and quality of life and to identify factors at the time of the diagnosis, that predict these ongoing symptoms.

### Patients

We conducted a cross-sectional multicenter cohort study among patients diagnosed with IIH during childhood between 2007 and 2012 at four medical centers: Bnai Zion, Schneider; Hillel Yaffe and Carmel. Patients were contacted in October 2024 to December 2024, at least 10 years after their initial diagnosis. Those who provided informed consent were interviewed by telephone. Data on IIH, along with demographic information, was retrieved from patients' medical records.

### Inclusion criteria

Inclusion criteria for the sample were as follows: diagnosis with IIH in childhood according to the revised diagnostic criteria published by Friedman (4); diagnosis and treatment details available in medical records; provision of informed consent to participate in the study; and willingness to complete the study questionnaires over the phone. There were no separate exclusion criteria. The study was approved by the local IRB BNZ 72-21.

## Methods

The telephone interview comprised of the Headache Impact Test (HIT)-6 questionnaire (10) to evaluate headache severity and a validated quality-of-life questionnaire, the WHOQOL-BREF (11), to assess the impact of headaches on daily life. The HIT-6 is a headache questionnaire with scores range from 36 to 78, with higher scores indicating more significant negative effects of headaches on daily life, categorized into four levels: Little/No Impact (≤49), Some Impact (50–55), Substantial Impact (56–59), and Severe Impact (60–78). Quality of life, was measured using the WHOQOL-BREF questionnaire. The WHOQOL-BREF is a 26-item, self-administered questionnaire developed by the World Health Organization (WHO) (11). It is an abbreviated version of the original WHOQOL-100 and is one of the most widely used instruments for measuring Quality of Life (QoL) across different cultures and health conditions. Unlike tools that focus strictly on symptoms or disease, the WHOQOL-BREF assesses an individual's perception of their position in life in the context of their culture and value systems. The questionnaire breaks down quality of life into four primary areas: (1) Physical Health: Energy and fatigue, sleep and rest, mobility, pain and discomfort, work capacity, and activities of daily living. (2) Psychological: Positive and negative feelings, self-esteem, body image, spirituality, and cognitive functions (memory/concentration). (3) Social Relationships: Personal relationships, social support, and sexual activity. (4) Environment: Financial resources, physical safety and security, health and social care (accessibility/quality), home environment, and opportunities for recreation. Items are rated on a 5-point Likert scale. Two items are examined separately: one asks about an individual's overall perception of quality of life, and the other asks about their overall perception of their health. The remaining 24 items are grouped into the four domains mentioned above. Scoring usually involves calculating a mean score for each domain and then transforming it into a scale from 0 to 100, where higher scores indicate a better quality of life.

### Statistical methods

Sample characteristics were described using means and standard deviations for continuous variables, and frequencies and percentages for categorical variables. Group differences for continuous variables with and without normal distribution were tested using the independent samples *t* test and Wilcoxon rank-sum test, respectively. Group differences for categorical data were tested using the chi-square test or Fisher's exact test, as appropriate, depending on whether the expected values were or were not above 5 in at least 80% of the cells. To evaluate correlation between two continuous variable we used the Pearson or Spearman correlation test for variables with and without normal distribution, respectively. All hypothesis tests were two-tailed and considered significant at a *p*-value <0.05. Analyses were conducted using R version (R Foundation for Statistical Computing, Vienna, Austria) in the RStudio Build environment (Posit PBC, Boston, MA, USA).

## Results

Out of 98 patients, 81 were recruited for the study (others could not be reached). Participants' mean age at IIH diagnosis was 13 (±2) years and mean age at follow-up was 24 (±5) years Sixty-six percent (54/81) were females and mean BMI was 27. Mean opening pressure was 39 mmH_2_O. Sixty-eight percent (43/63) of participants were treated with acetazolamide following their initial diagnosis and five (5/63 8%) underwent a lumboperitoneal shunt. Non received topiramate or furosemide. Data on medical treatment was missing for 18 patients. At the time of follow-up; none of the patients was treated with medication for IIH (acetazolamide/topiramate), and non-reported of psychiatric medical treatment. Fifty eight percent of our patients (47/81) were experiencing headaches at the time of follow-up (Table 1), with no significant difference between the sex. Relative to the no-headaches group, those with headaches at follow-up had initial higher BMI (32 vs. 25 *p* = 0.041) and lower quality of life (91 vs. 99 *p* = 0.017). The group with no headaches at follow-up had a significantly higher acetazolamide treatment rate (88% vs. 56%, *p* = 0.010). Within the group with headaches at follow-up, those with more significant headache burdens (HIT-6 levels 3–4) were older, had initial higher BMI, and had lower QoL (*p* = 0.020, *p* = 0.006, *p* = 0.010 respectively; Table 2) compared to those with lower headache burdens. Mean scores for the WHOQOL-BREF across the entire group were; 18.9 for Physical Health, 17.9 for Psychological health, 12.63 for Social Relationships and 12.5 for Environment. Analysis by headache severity showed a clear decline in total quality of life: the no-headache group scored 99, the mild group scored 97, and the substantial group scored 87. We found a positive correlation between HIT-6 level and initial BMI (0.638, *p* < 0.001), and negative correlations between QoL and both HIT-6 level (−0.519, *p* = 0.005) and BMI (−0.499, *p* = 0.007) (Table 3).

**Table 1:** Clinical features of patients with IIH, 10 years following their initial diagnosis.

Headache at follow-up	Headache, *N* = 47^a^	No headache, *N* = 34^a^	*p* value^b^
Age at onset (years)	14.4 (5.9)	11.4 (4.5)	0.056
Age at follow-up (years)	29 (14)	21 (6)	0.030
Sex (female)	34 (73%)	20 (59%)	0.3
BMI	32 (11)	25 (4)	0.041
Headache at diagnosis	36 (77%)	9 (26%)	0.013
Years to follow-up	14 (10)	11 (3)	0.3
Diplopia	4/21 (19%)	3 (9%)	0.4
Transient visual obscuration	5/22 (23%)	8 (24%)	0.8
Vomiting	12/44 (27%)	5 (15%)	0.2
Pulsatile tinnitus	1 (4.8%)	0 (0%)	0.4
QoL	91 (16)	99 (10)	0.017
Opening pressure (cmH_2_O)	39 (9)	38 (15)	0.3
Acetazolamide	22/39 (56%)	21/24 (88%)	0.010

IIH, idiopathic intracranial hypertension; QoL, quality of life.

a Mean (SD); *n*/*N* (%).

b Wilcoxon rank sum test; Pearson's chi-squared test; Fisher's exact test.

**Table 2:** HIT-6 levels and clinical features of patients with IIH, 10 years following their initial diagnosis.

HIT-6 level	No headache, *N* = 34^a^	Level 1–2, *N* = 18^a^	Level 3–4, *N* = 29^a^	*p* value^b^
Age at onset (years)	11.4 (4.5)	14.3 (6.5)	14.8 (5.9)	0.085
Age at follow-up (years)	21 (6)	26 (13)	31 (14)	0.020
Sex (female)	20 (59%)	11 (61%)	23 (79%)	0.15
BMI	25 (4)	25 (5)	35 (12)	0.006
Headache at diagnosis	9/34 (26%)	5/6 (83%)	5/7 (71%)	0.042
Years to follow-up	11 (3)	11 (7)	16 (11)	0.5
QoL	99 (10)	97 (11)	87 (17)	0.010
Acetazolamide	21/23 (91%)	8/15 (53%)	14/25 (56%)	0.002

a Mean (SD); *n*/*N* (%).

b Kruskal–Wallis rank sum test; Pearson's chi-squared test; Fisher's exact test.

**Table 3:** Computed correlations between HIT-6 scores, quality of life, and BMI among participants still experiencing headaches 10 years following their IIH initial diagnosis.^a^

Variables	Age at onset (years)	HIT-6	BMI	QoL
HIT-6	0.055 (.781)			
BMI	0.358 (.062)	0.638 (<.001)		
QoL	0.083 (.674)	−0.519 (.005)	−0.499 (.007)	
Years from diagnosis to follow-up	0.446 (.017)	0.195 (.319)	0.585 (.001)	0.129 (.513)

a Computed using Spearman's rank correlations with listwise deletion.

## Discussion

Our study highlights that headache remain prevalent years after the initial diagnosis of Idiopathic Intracranial Hypertension, impacting patients' quality of life. Even after resolution of elevated intracranial pressure, many patients continue to experience headaches, as reflected by HIT-6 scores, with most reporting a substantial effect on daily functioning. This persistent symptomatology suggests that the burden of IIH extends well beyond the acute phase, underscoring the need for a deeper understanding of its long-term management.

Our study is unique as a cross-sectional cohort long-term cohort follow-up of patients with IIH. We also used both the HIT-6 and a validated quality-of-life questionnaire (the WHOQOL-BREF) to glean valuable insights into patients' subjective experiences. The negative correlation found between headache severity and QoL shows that headaches have a real impact on patients' daily life and underscores the importance of addressing this symptom to improve patients' overall well-being.

McGirt et al. (12) in a retrospective long-term study on headache outcomes among patients with IIH, reported recurrence rates of 19% at 12 months and 48% at 36 months, despite properly functioning shunts after the initial placement surgery. The recurrence of headaches in that study is particularly notable because it occurs even though intracranial pressure was well-regulated and the shunt was functioning properly, suggesting that mechanisms other than pressure elevation may contribute to headache persistence. Yri (13) also reported a high rate of headache recurrence, but it was observed only after 12 months of follow-up.

In our study, we observed that a higher body mass index, at the time of diagnosis, was correlated with increased headache impact and reduced quality of life, at the follow-up time years ahead. Since elevated BMI is a recognized risk factor for IIH (1), our findings suggest that the recurrence of headaches in high-BMI patients, at the diagnosis, may be related to the underlying pathophysiology of IIH, even when intracranial pressure remains normal after many years. This finding implies that factors beyond increased pressure may play a role in headache persistence, highlighting the complex relationship between BMI and IIH in influencing headache severity and overall patient well-being (14). The relationship between headaches and obesity are complex. Body mass index (BMI) is strongly associated with several health-related challenges, including depression, reduced quality of life (QOL), poor exercise tolerance, and intracranial pressure. Individuals with frequent or severe headaches often struggle to maintain regular physical activity, which can lead to weight gain and further deterioration of both physical and mental health. Depression and chronic pain frequently coexist, and when combined with obesity, they contribute to a cycle of inactivity, emotional distress, and worsening symptoms. This cycle can significantly impact daily functioning and overall well-being. Elevated central adiposity may increase intra-abdominal pressure, which in turn raises central venous pressure and may impair normal cerebrospinal fluid dynamics. However, the exact mechanisms linking body mass to these symptoms are still being explored and likely involve multiple pathways.

Managing excess weight in these patients is essential but requires more than standard lifestyle advice. A comprehensive approach that includes medical, psychological, and physical activity interventions is often necessary. The intricate connection between BMI and symptom burden highlights the need for further research and more personalized treatment strategies.

The pathophysiology of headaches in Idiopathic Intracranial Hypertension syndrome, is multifaceted and probably extends beyond the elevated intracranial pressure direct effect (ICP). While increased ICP is a hallmark of IIH, leading to the compression of pain-sensitive structures such as the dura mater and cranial nerves, the persistence of headaches even after ICP normalization suggests additional mechanisms at play (4).

One theory might be central sensitization, where prolonged activation of pain pathways during the acute phase results in heightened sensitivity within the trigeminal vascular system. This heightened sensitivity can cause headaches that persist independently of the ICP levels. Additionally, obesity—a recognized risk factor for IIH—is associated with chronic low grade systemic inflammation, driven by adipose tissue secretion of pro-inflammatory cytokines such as TNF-α, IL-6. This inflammatory milieu may promote neuroinflammation, exacerbating pain perception and sustaining headache symptoms (15). Leptin, which is elevated in obesity, has been linked to altered hypothalamic regulation and may contribute to pathophysiology (2). In women, higher BMI is also linked to elevated estradiol and testosterone levels, which may influence CSF dynamics and further increase susceptibility to persistent headaches in patients with IIH (16).

Vascular factors also play a role; impaired cerebral venous outflow can lead to venous congestion, resulting in increased venous pressure and decreased cerebrospinal fluid absorption. This venous hypertension may perpetuate elevated ICP and contribute to headache persistence (17).

Furthermore, psychological components, including anxiety and depression, can lower pain thresholds and amplify the subjective experience of headaches, creating a biopsychosocial feedback loop that perpetuates symptoms (18).

Recent studies have highlighted that even after normalization of ICP, most patients continue to experience persistent headaches, indicating that factors beyond ICP contribute to headache pathophysiology in IIH (19, 20).

Understanding these diverse mechanisms is crucial for developing comprehensive treatment strategies that address not only ICP reduction but also central sensitization, systemic inflammation, vascular health, and psychological well-being to effectively manage and alleviate persistent headaches in patients with IIH.

### Limitations

For practical reasons, data were collected via telephone interviews. Consequently, we were unable to conduct a comprehensive clinical evaluation, including in-person neurological and ophthalmological examinations. As a result, key physical signs of IIH, such as papilledema could not be assessed, and intracranial pressure, a crucial sign of disease severity- could not be directly measured. Because no repeat lumbar puncture was performed, we cannot determine with certainty whether the headache reported are related to elevated intracranial pressure or is occurred independently. BMI levels were reported among children only as a raw number and were not adjusted for age and sex. These limitations may affect the accuracy of symptom reporting and the generalizability of our results. Furthermore, most participants were within the typical childbearing age range for IIH at the time of follow-up; therefore, our findings regarding persistent headaches may not apply to older individuals.

## Conclusion

In conclusion, a substantial proportion of patients with IIH continue to experience headaches many years after their initial diagnosis, with a significant negative impact on their quality of life. This persistent symptom burden underscores the need for long-term follow-up, enabling IIH ongoing monitoring, early detection of recurrence and timely interventions. Future research is needed to clarify the mechanisms driving headache in IIH and to develop targeted, effective treatments for this challenging complication.

## Data Availability

The datasets presented in this article are not readily available because technical. Requests to access the datasets should be directed to Jacob Genizi genizij@gmail.com.
